# Medication adherence is associated with the necessity-concern differential in newly diagnosed primary Sjögren disease: a prospective observational cohort study

**DOI:** 10.1007/s00296-026-06201-5

**Published:** 2026-08-22

**Authors:** Kemal Erol, Ezgi Akyıldız Tezcan, Naciye Baş Bilen, Cemal Gürbüz

**Affiliations:** 1 Division of Rheumatology, Department of Physical Medicine and Rehabilitation, Faculty of Medicine, Selçuk University, Konya, Turkey; 2 Department of Physical Medicine and Rehabilitation, Faculty of Medicine, Selçuk University, Konya, Turkey

**Keywords:** Sjogren’s Syndrome, Medication adherence, Surveys and questionnaires, Patient reported outcome measures, Prospective studies, Cohort studies

## Abstract

The objectives of this study were, firstly, to assess medication adherence and, secondly, to evaluate the relationships between medication adherence and symptoms, disease activity, and patient-reported outcomes in patients with newly diagnosed primary Sjögren disease (pSjD). In this prospective observational cohort study, 54 patients with newly diagnosed pSjD were enrolled in a tertiary rheumatology clinic. Forty patients (74.1%) were reassessed after a mean follow-up of 21.36 ± 4.79 months. Medication adherence was evaluated using the Compliance Questionnaire for Rheumatology-5 (CQR-5), classifying patients as low or high adherence. Disease activity, symptom burden, psychological symptoms, fatigue, functional status, health-related quality of life, and medication beliefs were evaluated using ESSDAI, ESSPRI, HADS, fatigue severity assessment, HAQ, SF-12, and the Beliefs about Medicines Questionnaire (BMQ). The necessity-concern differential (NCD) was calculated as the BMQ-specific necessity score minus the concern score. Logistic regression analysis was exploratory. The cohort was predominantly female (52/54, 96.3%), with a mean age of 49.7 ± 9.5 years. At follow-up, 16 patients (40%) were classified as low adherence and 24 (60%) as high adherence. Baseline clinical and sociodemographic characteristics were comparable between groups. ESSDAI scores remained low and stable over time, and ESSPRI increased modestly in both groups without significant between-group differences. Psychological status, fatigue, functional status, and quality of life were similar across adherence categories. Specific necessity scores were numerically higher in high-adherence patients (3.37 ± 0.79 vs. 3.04 ± 1.06; *p* = 0.267), whereas concern scores tended to be higher in low-adherence patients (3.46 ± 0.92 vs. 3.07 ± 0.63; *p* = 0.115). The NCD was significantly lower in the low-adherence group than in the high-adherence group (− 0.43 ± 1.16 vs. 0.30 ± 0.96; *p* = 0.038). In exploratory logistic regression, higher NCD was associated with high adherence (OR 1.95, 95% CI 1.01–3.76). In patients with newly diagnosed pSjD, low medication adherence was frequent and was associated with patients’ necessity-concern balance rather than clinical disease activity or patient-reported burden. Because adherence and medication beliefs were assessed at the same time point and CQR-5 has not been specifically validated in pSjD, these findings should be interpreted as exploratory and non-causal.

**Supplementary Information:**

The online version contains supplementary material available at https://doi.org/10.1007/s00296-026-06201-5.

## Introduction

Sjögren disease (SjD) is a chronic systemic autoimmune disorder characterized by lymphocytic infiltration of exocrine glands, leading to xerostomia and keratoconjunctivitis sicca. In addition to glandular involvement, the disease may affect multiple extraglandular systems, including musculoskeletal, cutaneous, gastrointestinal, pulmonary, cardiac, endocrine, renal, hematologic, obstetric, and nervous systems [1, 2]. When SjD occurs in isolation, it is referred to as primary Sjögren disease (pSjD), whereas its coexistence with other autoimmune disorders such as systemic lupus erythematosus, rheumatoid arthritis, or systemic sclerosis is defined as associated Sjögren disease [3]. pSjD is among the most common autoimmune diseases, with an estimated prevalence ranging from 0.06 to 1.5%. It predominantly affects women, with a female-to-male ratio of approximately 10:1, and typically presents in the fifth to sixth decades of life [4]. Beyond glandular symptoms, pSjD is associated with a substantial burden of fatigue, pain, depression, anxiety, and sleep disturbances, all of which contribute to impaired quality of life [2].

Medication adherence, defined as the extent to which a patient’s medication-taking behavior corresponds with agreed recommendations, is a key component of effective management in chronic rheumatic diseases. Suboptimal adherence has been consistently associated with poorer clinical outcomes, including higher disease activity, worse functional status, and increased healthcare utilization [5, 6]. In rheumatoid arthritis, nonadherence rates have been reported to range from 30 to 80%, depending on the assessment method, and have been linked to reduced treatment response and impaired long-term disease control [7]. Similarly, in systemic lupus erythematosus, between one-quarter and more than half of patients are considered nonadherent, a behavior associated with disease flares, organ damage, and reduced quality of life [8, 9]. Comparable patterns have been reported in axial spondyloarthritis, where approximately one-third to one-half of patients do not fully adhere to prescribed therapies, and patients’ illness perceptions and beliefs about medicines have been shown to be associated with adherence behavior [10, 11]. Recent publications have also emphasized that adherence-related problems are relevant across rheumatic diseases, including treatment compliance and persistence with biological therapies in rheumatoid arthritis and the association between medication adherence and health empowerment in gout [12, 13].

Despite the increasing recognition of adherence as an important aspect of disease management across rheumatology, data on medication adherence in pSjD remain limited. Available evidence is sparse and largely indirect. For example, a recent study evaluating hydroxychloroquine blood levels suggested that a considerable proportion of patients had subtherapeutic concentrations, indicating potentially suboptimal adherence [14]. However, prospective studies using patient-reported adherence measures and simultaneously evaluating clinical, psychological, and perceptual factors—particularly in prospective cohorts—are lacking.

In addition, it is important to distinguish between newly diagnosed disease and early disease, as symptom onset in SjD may precede diagnosis by several years. Therefore, patients classified as newly diagnosed may still represent a heterogeneous group in terms of disease duration and burden.

While the relationship between medication beliefs and adherence has been studied in several rheumatic diseases, data addressing this relationship specifically in pSjD are scarce, particularly in newly diagnosed cohorts in which adherence, medication beliefs, systemic disease activity, Sjögren-specific symptoms, and patient-reported outcomes are evaluated together.

Accordingly, the present study aimed to evaluate medication adherence during follow-up in patients with newly diagnosed pSjD and to examine its associations with clinical characteristics, patient-reported outcomes, and beliefs about medicines.

## Materials and methods

This prospective cohort study enrolled 54 consecutive patients aged 18–65 years who received a new diagnosis of pSjD in a tertiary hospital’s rheumatology outpatient clinic. All participants fulfilled the 2016 American College of Rheumatology/European Alliance of Associations for Rheumatology (ACR/EULAR) classification criteria [15].

Patients were reassessed after a mean follow-up of 21.36 ± 4.79 months. Of the initial cohort, 40 patients (74.1%) attended the follow-up assessment. Of these, 25 patients (46.3%) attended the follow-up visit as scheduled, whereas 15 patients (27.8%) were contacted by telephone and subsequently invited for an in-person follow-up visit. Baseline characteristics of patients who completed follow-up and those who were lost to follow-up were compared to assess potential selection bias.

**Reporting guideline:** This prospective observational cohort study was reported in accordance with the Strengthening the Reporting of Observational Studies in Epidemiology (STROBE) statement for cohort studies. The completed STROBE checklist was prepared and submitted as Supplementary File 1 [16].

This study was approved by the Selçuk University Rectorate Local Ethics Committee (approval no: 2024/296; meeting date: 04 June 2024; official document date and number: 05 June 2024, E-70632468-050.04-768772). Written informed consent was obtained from all participants. The study was conducted in accordance with the Declaration of Helsinki, October 2024 version, and Good Clinical Practice principles.

Baseline demographic and clinical characteristics were recorded using a structured questionnaire, including age, sex, height, weight, smoking status, symptom duration, employment, and educational level. It should be noted that “newly diagnosed” refers to the time of diagnosis rather than symptom onset; therefore, symptom duration was recorded to better characterize disease stage.

Systemic disease activity was evaluated at baseline and at follow-up using the EULAR Sjögren’s Syndrome Disease Activity Index (ESSDAI), which assesses 12 organ domains: constitutional, lymphadenopathy, glandular, articular, cutaneous, pulmonary, renal, peripheral and central nervous system, muscular, hematological, and biological involvement. Total scores range from 0 to 123 and are categorized as low (< 5), moderate [5–13], or high (≥ 14) [17].

Patient-reported symptoms were assessed using the EULAR Sjögren’s Syndrome Patient Reported Index (ESSPRI), which evaluates dryness, pain, and fatigue on 0–10 numerical rating scales, and the total score is calculated as the mean of these domains [18].

Medication adherence was assessed at the follow-up visit using the 5-item Compliance Questionnaire for Rheumatology (CQR-5), a brief self-report instrument developed to identify patients at risk of suboptimal adherence [19]. The Turkish version of the CQR-5 has demonstrated good validity and reliability in patients with rheumatoid arthritis [20]; however, its use in patients with pSjD has not been specifically validated and should be interpreted with caution. Items are rated on a Likert scale, and according to recommended scoring procedures, patients were categorized into low- and high-adherence groups.

Patients’ beliefs regarding their prescribed treatments were evaluated at the same follow-up time point using the Beliefs about Medicines Questionnaire (BMQ), which includes specific necessity and concern subscales. This instrument was originally developed by Horne and colleagues to assess cognitive representations of medication and has been widely used and adapted in various patient populations [21]. Higher necessity scores indicate stronger beliefs in the need for medication, whereas higher concern scores reflect apprehension about potential adverse effects. The necessity-concern differential (NCD) was calculated by subtracting the concern score from the necessity score. As adherence and beliefs were assessed at the same time point, analyses reflect associations rather than temporal or predictive relationships.

**Questionnaire-based assessments:** The questionnaire-based assessments were planned, administered, and reported with reference to current recommendations for survey and questionnaire-based research. All patient-reported instruments were administered according to their published instructions and using validated versions where available. The timing of each assessment was prespecified: ESSPRI, HADS, fatigue severity assessment, HAQ, and SF-12 were evaluated at baseline and follow-up, whereas medication adherence using the CQR-5 and beliefs about medicines using the BMQ were assessed at the follow-up visit. The CQR-5 was used to classify patients into low- and high-adherence groups as a brief self-report adherence screening instrument. Although the Turkish version of the CQR-5 has demonstrated validity and reliability in rheumatoid arthritis, it has not been specifically validated in primary Sjögren disease; therefore, CQR-5-based adherence classification was interpreted cautiously. The BMQ was used to assess patients’ perceived necessity of treatment and concerns about medicines, and the necessity-concern differential was calculated by subtracting the concern score from the necessity score. Because adherence and medication beliefs were measured at the same follow-up time point, these analyses were considered cross-sectional within the follow-up assessment and were interpreted as associations rather than temporal or causal relationships. The questionnaires and scales were used in accordance with their published instructions, validated versions, and applicable academic-use or licensing requirements; no full questionnaire forms or copyrighted item content are reproduced in this manuscript [22].

Patients were managed according to routine clinical practice. Detailed information regarding specific medications, number of treatments, treatment modifications, or adverse effects was not systematically collected as part of the study protocol; therefore, medication adherence was evaluated independently of specific treatment regimens.

Psychological symptoms were assessed using the Hospital Anxiety and Depression Scale (HADS). Functional status was evaluated with the Health Assessment Questionnaire (HAQ), and health-related quality of life was measured using the 12-item Short Form Health Survey (SF-12). All measures were recorded at baseline and at follow-up.

All statistical analyses were performed using IBM SPSS Statistics version 22 (IBM Corp., Armonk, NY, USA). The Shapiro–Wilk test and visual inspection of histograms and Q–Q plots were used to assess the normality of continuous variables. Normally distributed variables are presented as mean ± standard deviation, whereas non-normally distributed variables are reported as median with interquartile range. Categorical variables are expressed as numbers and percentages.

Between-group comparisons were performed according to the distribution and type of variables. The independent-samples t-test or Mann–Whitney U test was used for continuous variables, and the chi-square test or Fisher’s exact test was used for categorical variables, as appropriate. Within-group changes from baseline to follow-up were assessed using paired-samples t-tests for normally distributed variables and Wilcoxon signed-rank tests for non-normally distributed variables. In addition to p values, numerical differences between groups, direction of change, odds ratios, 95% confidence intervals, and model-fit indices were considered when interpreting the findings.

Binary logistic regression analysis was performed to explore factors associated with high medication adherence. Because of the relatively small number of patients and events, the regression analysis was intentionally restricted and considered exploratory. The necessity-concern differential was entered as the main explanatory variable because it represented the principal perceptual construct of interest and showed the clearest between-group separation in univariable analyses. No multivariable overfitted model was constructed. Model fit was evaluated using the omnibus test, Cox and Snell R^2^, Nagelkerke R^2^, and overall classification accuracy. All tests were two-sided. A p value < 0.05 was considered statistically significant; however, statistical significance was not interpreted as evidence of causality.

## Results

### Patient characteristics and adherence classification

A total of 54 patients with newly diagnosed pSjD were enrolled. The mean age was 49.7 ± 9.5 years, and 52 patients (96.3%) were female. At follow-up, 40 patients (74.1%) were available for reassessment. The mean follow-up duration was 21.36 ± 4.79 months.

Among reassessed patients, the CQR-5 identified 16 (40.0%) as having low adherence and 24 (60.0%) as having high adherence.

Baseline characteristics of patients who completed follow-up and those lost to follow-up were comparable, with no significant differences in age, sex, body mass index, or symptom duration (all *p* > 0.05).

### Sociodemographic characteristics

Baseline characteristics according to adherence status are presented in Table 1. Age (*p* = 0.971), body mass index (*p* = 0.380), and symptom duration (*p* = 0.279) were similar between the low- and high-adherence groups. Sex distribution, employment status, smoking habits, and education level also did not differ significantly between groups.

**Table 1:** Baseline sociodemographic characteristics according to the CQR-5 adherence group

	Low adherence (n:16)	High adherence (n:24)	p-value
Age, years, mean (SD)	49.94 (8.44)	50.04 (9.27)	0.971^a^
BMI, kg/m^2^, mean (SD)	30.29 (5.99)	28.68 (5.33)	0.380^a^
Symptom duration, years, median (25th–75th)	3.5 (2.0–8.75)	2.0 (1.0–5.0)	0.279^b^
Sex, n (%) female	15 (93.8%)	24 (100.0%)	0.400^c^
Employment status, n (%) not active	13 (81.2%)	21 (87.5%)	0.668^c^
Smoking, n (%) non-smoker	14 (87.5%)	22 (91.7%)	0.999^c^
Education, n (%) high school graduate or above	4 (25.0%)	6 (25.0%)	0.999^c^

The predominance of female participants should be considered when interpreting between-group comparisons

Some variables (e.g., education level) showed limited variability between groups

*BMI* Body mass index, *CQR-5* Compliance Questionnaire on Rheumatology-5, *kg/m*^*2*^ kilogram per square meter, *n* number, *SD* Standard deviation

^a^Independent-samples t-test

^b^Mann–Whitney U test

^c^Fisher’s exact test

### Disease activity and Sjögren-specific symptom burden

#### Cross-sectional comparisons

Baseline and follow-up ESSDAI and ESSPRI values are presented in Table 2, and categorical distributions are shown in Table 3. Systemic disease activity was low in both adherence groups at baseline and remained low at follow-up, with no significant between-group differences (baseline *p* = 0.621; follow-up *p* = 0.736).

**Table 2:** ESSDAI and ESSPRI scores at baseline and Follow-up according to the CQR-5 adherence status

	Time point	Low adherence (n:16)	High adherence (n:24)	p-value
ESSDAI score, median (25th–75th percentile)	Baseline	1.00 (0.00–8.75)	1.00 (0.00–3.75)	0.733^a^
	Follow-up	1.50 (0.00–8.75)	1.00 (0.00–4.00)	0.452^a^
	Change (follow-up–baseline)	0.0 (0.0–0.0)	0.0 (− 0.75–0.0)	0.469^a^
ESSPRI total score	Baseline, mean (SD)	5.97 (2.41)	5.34 (2.08)	0.389^b^
	Follow-up, median (25th–75th percentile)	7.32 (6.08–8.17)	6.33 (5.41–7.58)	0.134^a^
	Change (follow-up–baseline), mean (SD)	0.85 (2.52)	0.92 (1.95)	0.918^b^

Continuous variables are presented according to distribution

*CQR 5* Compliance Questionnaire on Rheumatology (5-item version), *ESSDAI* EULAR Sjögren’s Syndrome Disease Activity Index, *ESSPRI* EULAR Sjögren’s Syndrome Patient-Reported Index, *SD* Standard deviation

^a^Mann–Whitney U test

^b^Independent-samples t-test

**Table 3:** ESSDAI and ESSPRI severity categories at baseline and Follow-up according to the CQR-5 adherence status

	Category	Low adherence n (%)	High adherence n (%)	*p*-value
ESSDAI baseline	Low (0–4)	11 (68.8)	20 (83.3)	0.621^a^
	Moderate (5–13)	3 (18.8)	3 (12.5)	
	High (≥ 14)	2 (12.5)	1 (4.2)	
ESSDAI Follow-up	Low (0–4)	11 (68.8)	19 (79.2)	0.736^a^
	Moderate (5–13)	3 (18.8)	4 (16.7)	
	High (≥ 14)	2 (12.5)	1 (4.2)	
ESSPRI baseline	Low (0–4)	4 (25.0)	10 (41.7)	0.279^b^
	High (≥ 5)	12 (75.0)	14 (58.3)	
ESSPRI Follow-up	Low (0–4)	2 (12.5)	2 (8.3)	0.999^c^
	High (≥ 5)	14 (87.5)	22 (91.7)	

*CQR 5* Compliance Questionnaire on Rheumatology (5-item version), *ESSDAI* EULAR Sjögren’s Syndrome Disease Activity Index, *ESSPRI* EULAR Sjögren’s Syndrome Patient-Reported Index, *SD* Standard deviation

^a^Fisher’s exact test (Monte Carlo simulation)

^b^Pearson chi-square test

^c^Fisher’s exact test

ESSPRI scores were also comparable between groups. At baseline, total scores and the proportion of patients with high symptom burden did not differ significantly (*p* = 0.389 and *p* = 0.279, respectively). Similar findings were observed at follow-up (*p* = 0.134 and *p* = 0.999, respectively).

#### Longitudinal analyses

ESSDAI remained stable over time in both groups. In the low-adherence group, median values changed from 1.00 (0.00–8.75) to 1.50 (0.00–8.75) (*p* = 0.999). In the high-adherence group, ESSDAI remained at 1.00 (*p* = 0.366).

ESSPRI increased in both groups. In low-adherence patients, scores rose from 5.97 ± 2.41 to 6.82 ± 1.81 (mean change + 0.85, *p* = 0.197). In high-adherence patients, ESSPRI increased from 5.34 ± 2.08 to 6.24 ± 1.64 (mean change + 0.90, *p* = 0.032).

However, between-group comparisons of change scores did not show statistically significant differences. Median ESSDAI change was 0.0 (0.0–0.0) versus 0.0 (− 0.75 to 0.0) (p = 0.469), and mean ESSPRI change was 0.85 ± 2.52 versus 0.92 ± 1.95 (*p* = 0.918).

### Psychological symptoms, fatigue, functional status, and health-related quality of life

Baseline and follow-up data are summarized in Table 4. At baseline, SF-12 physical, mental, and total scores did not differ significantly between low- and high-adherence patients (*p* = 0.484, 0.405, and 0.415, respectively). HADS anxiety and depression scores were also similar (*p* = 0.821 and *p* = 0.580, respectively). Fatigue severity and HAQ scores showed no significant differences (*p* = 0.902 and *p* = 0.090, respectively).

**Table 4:** Baseline and follow-up psychological symptoms, fatigue, functional status, and SF-12 scores according to the CQR-5 adherence group

	Low adherence (n:16)	High adherence (n:24)	*p*-value
SF-12 physical component, mean (SD)	33.44 (18.92)	38.08 (21.24)	0.484^a^
SF-12 mental component, mean (SD)	44.00 (17.78)	49.58 (22.18)	0.405^a^
SF-12 total score, mean (SD)	39.81 (17.21)	44.96 (20.62)	0.415^a^
Fatigue severity, median (25th–75th percentile)	5.44 (3.08–6.08)	5.74 (3.49–6.45)	0.902^b^
HAQ, median (25th–75th percentile)	0.63 (0.16–1.09)	0.22 (0.00–0.63)	0.090^b^
HADS-Anxiety, mean (SD)	8.13 (4.96)	8.50 (5.20)	0.821^a^
HADS-Depression, mean (SD)	7.69 (5.03)	8.54 (4.55)	0.580^a^
SF-12 physical component (follow-up), mean (SD)	39.81 (18.13)	42.63 (23.89)	0.692^a^
SF-12 mental component (follow-up), mean (SD)	53.31 (16.97)	51.67 (14.59)	0.745^a^
SF-12 total score (follow-up), mean (SD)	48.38 (17.46)	47.50 (17.57)	0.878^a^
Fatigue severity (follow-up), median (25th–75th percentile)	6.30 (4.13–7.00)	6.10 (4.91–6.95)	0.774^b^
HAQ (follow-up), median (25th–75th percentile)	0.63 (0.00–1.00)	0.75 (0.28–1.50)	0.113^b^
HADS-Anxiety (follow-up), mean (SD)	7.38 (3.05)	7.96 (4.59)	0.658^a^
HADS-Depression (follow-up), mean (SD)	8.31 (3.30)	8.17 (4.28)	0.909^a^

*SF-12* 12-Item Short Form Health Survey, *HADS* Hospital Anxiety and Depression Scale, *HAQ* Health Assessment Questionnaire, *CQR-5* 5-item Compliance Questionnaire on Rheumatology, *HRQoL* health-related quality of life

^a^Independent-samples t-test

^b^Mann–Whitney U test

At follow-up, results remained comparable across groups. SF-12 scores (*p* = 0.692, 0.745, and 0.878), HADS anxiety and depression scores (*p* = 0.658 and *p* = 0.909), fatigue (*p* = 0.774), and HAQ (*p* = 0.113) were not significantly different between groups.

### Longitudinal changes in patient-reported outcomes

SF-12 scores showed numerical improvement in both groups but did not reach statistical significance. In the low-adherence group, changes were + 6.38, + 9.31, and + 8.56 (*p* = 0.272, *p* = 0.102, and *p* = 0.127), while in the high-adherence group, changes were + 4.54, + 2.08, and + 2.54 (*p* = 0.362, *p* = 0.621, and *p* = 0.521).

HADS anxiety decreased slightly in both groups (− 0.75 vs − 0.54; *p* = 0.509 and *p* = 0.712), and depression scores showed minimal variation (+ 0.63 vs − 0.38; *p* = 0.703 and *p* = 0.785). Fatigue increased modestly (+ 0.68 vs + 0.63; *p* = 0.169 and *p* = 0.064). HAQ scores changed by − 0.15 (*p* = 0.495) and + 0.43 (*p* = 0.062) in low- and high-adherence groups, respectively.

Between-group comparisons of change scores did not reveal statistically significant differences for any of these outcomes.

### Beliefs about medicines

Specific necessity scores were numerically higher in the high-adherence group (3.37 ± 0.79) compared with the low-adherence group (3.04 ± 1.06), but this difference did not reach statistical significance (*p* = 0.267). Specific concerns tended to be higher in low-adherence patients (3.46 ± 0.92 vs 3.07 ± 0.63, *p* = 0.115). General harm beliefs were similar between groups (*p* = 0.530), and general overuse beliefs did not differ significantly (*p* = 0.062).

In contrast, the necessity-concern differential (NCD) differed significantly between groups, with lower values observed in low-adherence patients (− 0.43 ± 1.16) compared with high-adherence patients (0.30 ± 0.96; *p* = 0.038). Although the absolute difference in the necessity-concern differential was modest, its direction was consistent with the prespecified conceptual framework: patients with lower adherence had a less favorable balance between perceived necessity and medication-related concerns.

### Logistic regression

In the exploratory binary logistic regression model, the necessity-concern differential was entered as the main explanatory variable for high medication adherence. The model showed statistically significant overall fit compared with the null model (Omnibus *χ*^2^ = 4.46, *p* = 0.035). Each one-point increase in the necessity-concern differential was associated with higher odds of being classified in the high-adherence group (OR = 1.95, 95% CI 1.01–3.76). The model explained a modest proportion of variance, with Cox and Snell R^2^ of 10.6% and Nagelkerke R^2^ of 14.3%, and had an overall classification accuracy of 65%. Given the small sample size and the cross-sectional assessment of adherence and medication beliefs at follow-up, this model was interpreted as hypothesis-generating rather than confirmatory.

## Discussion

In this prospective cohort study of patients with newly diagnosed pSjD, we observed substantial variability in medication adherence during follow-up. Nearly one quarter of the initial cohort did not attend the planned follow-up visit, and among those reassessed, 40% were categorized as having low adherence according to the CQR-5. Importantly, patients with low and high adherence were comparable in terms of baseline clinical and sociodemographic characteristics. In contrast, adherence was associated with how patients balanced the perceived necessity of their medication against concerns about potential harm, underscoring the relevance of cognitive and perceptual factors in treatment behavior.

The frequency of suboptimal adherence observed in our study is broadly consistent with findings reported in other rheumatic diseases. In rheumatoid arthritis, nonadherence rates ranging from 30 to 80% have been described depending on assessment methods [7]. In systemic lupus erythematosus, approximately one quarter to more than half of patients are considered nonadherent [8, 9]. Similar patterns have been reported in axial spondyloarthritis, where patients’ illness perceptions and medication beliefs have been shown to be associated with adherence [10, 11]. In contrast, evidence on medication adherence in pSjD remains limited. A recent study evaluated hydroxychloroquine blood concentrations in patients with Sjögren disease and reported that a considerable proportion of patients had subtherapeutic levels, indirectly suggesting suboptimal adherence [14]. Unlike that study, which focused on a single medication and used pharmacological monitoring, the present study evaluated self-reported adherence together with medication beliefs, disease activity, Sjögren-specific symptom burden, and broader patient-reported outcomes. Although self-reported adherence measures may overestimate true adherence compared with objective methods [23], our findings suggest that adherence-related challenges are also relevant in patients with newly diagnosed pSjD.

Although the relationship between medication beliefs and adherence has been investigated in other rheumatic diseases, data specific to pSjD are scarce. This distinction is clinically relevant because pSjD differs from other inflammatory rheumatic diseases in terms of treatment goals, the frequent coexistence of sicca symptoms and fatigue, often low systemic disease activity, and heterogeneous use of symptomatic and immunomodulatory therapies. In the present study, adherence status was not clearly associated with ESSDAI, ESSPRI, psychological symptoms, fatigue, functional status, or health-related quality of life, whereas the necessity-concern differential differed between low- and high-adherence groups. Therefore, our findings extend the existing adherence literature by showing that the necessity-concern framework, previously described in other chronic rheumatic diseases, may also be relevant in pSjD. However, because adherence and medication beliefs were assessed at the same follow-up time point, this association should be interpreted as exploratory and non-causal.

Recent studies have shown that adherence-related problems are relevant across rheumatic diseases. Neycheva et al. [12] reported declining compliance with biological therapies over 36 months in rheumatoid arthritis, while Wang et al. [13] showed that health empowerment was associated with medication adherence in gout. Although these studies differ from ours in disease population and adherence assessment, they support the broader view that medication-taking behavior is shaped not only by disease-related variables but also by treatment context and patient-level factors. Our study adds pSjD-specific data by evaluating self-reported adherence together with medication beliefs, the necessity-concern differential, disease activity, and patient-reported outcomes.

We did not observe significant associations between adherence and age, body mass index, symptom duration, sex, educational level, smoking status, or employment. This pattern is consistent with observations from rheumatoid arthritis and lupus cohorts, in which sociodemographic variables alone have generally shown limited explanatory value [7, 8]. These findings further support the notion that demographic characteristics may be less informative than patients’ perceptions and interpretations of their illness and treatment.

Systemic disease activity, as measured by the ESSDAI, remained low and stable in both adherence groups and was not associated with adherence status. Similarly, symptom burden, fatigue, psychological status, functional ability, and health-related quality of life were comparable between groups both at baseline and over time. These findings suggest that, in newly diagnosed pSjD, adherence behavior may not be adequately explained by measurable clinical severity or patient-reported symptom burden. The generally low level of systemic activity in our cohort may also have limited the ability to detect potential relationships between adherence and clinical outcomes.

Beliefs about medicines showed the most consistent association with adherence in this cohort. Although the necessity and concern subscales did not differ significantly when considered separately, the necessity-concern differential was lower among patients with low adherence. This observation is in line with the Necessity-concerns Framework proposed by Horne and colleagues [21], which posits that adherence behavior reflects a balance between perceived personal need for treatment and concerns about potential adverse effects. In exploratory logistic regression, higher necessity-concern differential values were associated with higher odds of being classified as adherent. However, the effect estimate had a wide confidence interval and the model explained only a modest proportion of adherence variability. Therefore, this finding should be interpreted as a hypothesis-generating association rather than as evidence of an independent predictor or causal determinant of adherence.

From a clinical perspective, these findings suggest that evaluation of disease activity alone is insufficient to understand medication-taking behavior. Assessment of patients’ perceived need for treatment and medication-related concerns may help clinicians identify individuals who could benefit from more detailed treatment discussions. However, whether interventions targeting medication beliefs can improve adherence in pSjD requires confirmation in larger longitudinal and interventional studies.

### Limitations

This study has several limitations that should be considered when interpreting the findings. First, the sample size was relatively small, particularly after loss to follow-up, which may have limited statistical power and increased the risk of type II error. Second, the predominance of female participants resulted in a limited ability to assess sex-related differences and may restrict generalizability. Third, medication adherence was assessed using a self-report instrument, which may be subject to recall bias and social desirability bias and may overestimate true adherence compared with objective measures.

In addition, detailed information regarding specific medications, number of treatments, treatment modifications, and adverse effects was not systematically collected. Therefore, adherence could not be evaluated in relation to treatment characteristics, which may represent an important contextual factor. Furthermore, the CQR-5 has been validated in rheumatoid arthritis populations, and although widely used in inflammatory rheumatic diseases, its performance in pSjD has not been specifically established.

Another important limitation is that medication adherence and beliefs about medicines were assessed at the same time point. As a result, the observed associations should not be interpreted as indicating a temporal or causal relationship. Similarly, logistic regression analyses were performed in a relatively small sample and explained a limited proportion of variance; therefore, these findings should be considered exploratory. To avoid model overfitting, the analysis was deliberately restricted, and extensive multivariable adjustment was not attempted. The wide confidence interval around the odds ratio indicates statistical imprecision. Accordingly, the regression findings should be regarded as exploratory and hypothesis-generating and require confirmation in larger cohorts with prespecified multivariable models.

Finally, although patients were classified as newly diagnosed, symptom duration indicated that some individuals may have had a longer pre-diagnostic disease course. This heterogeneity may have influenced both clinical presentation and adherence behavior.

## Conclusion

In this prospective observational cohort of patients with newly diagnosed pSjD, low medication adherence was frequent during follow-up. Adherence was not clearly associated with systemic disease activity, Sjögren-specific symptom burden, psychological status, fatigue, functional status, or health-related quality of life. In contrast, a lower necessity-concern differential was observed among patients with low adherence and was associated with adherence status in exploratory logistic regression. Because medication adherence and beliefs about medicines were assessed at the same time point, and because the regression model was based on a relatively small sample, these findings should be interpreted as exploratory and non-causal. These findings highlight the potential importance of perceptual factors in adherence behavior. Future studies involving larger, multicenter cohorts, objective adherence measures, and longitudinal designs are needed to better clarify these relationships and to inform the development of targeted adherence-support strategies.

## Supplementary Information

Below is the link to the electronic supplementary material.

Supplementary Material 1

## Data Availability

The datasets generated and/or analyzed during the current study were not deposited in a public repository. Data are available from the corresponding author upon reasonable request.

## Declarations

### Use of artificial intelligence

Artificial intelligence-assisted tools were used only for language editing and improvement of grammar and readability. All AI-assisted edits were critically reviewed, verified, and approved by the authors. No artificial intelligence tool was used to generate original data, perform statistical analyses, interpret the results, or draw scientific conclusions.

### Permission to use questionnaires

The questionnaires and scales used in this study were administered in accordance with their published instructions, validated versions, and applicable academic-use or licensing requirements. No full questionnaire forms or copyrighted item content are reproduced in this manuscript.

### Related congress abstract publication

This work has not been previously presented or published as a congress abstract.

### Disclaimer

No part of this manuscript has been copied from or published elsewhere, in whole or in part.
