# Supplementary material for: Medication adherence is associated with the necessity-concern differential in newly diagnosed primary Sjögren disease: a prospective observational cohort study
Source: Rheumatol Int. 2026 Aug 22;46(9):242. doi: 10.1007/s00296-026-06201-5 (PMC13499819; doi:10.1007/s00296-026-06201-5)
Supplement: Supplementary file 1 [file 296_2026_6201_MOESM1_ESM.docx]

**STROBE Statement—Checklist of Items That Should Be Included in Reports of Cohort Studies**

**Supplementary File 1**

| **Manuscript number** | RHEI-D-26-00651 |
| --- | --- |
| **Journal** | Rheumatology International |
| **Manuscript title** | Medication Adherence Is Associated with the Necessity–Concern Differential in Newly Diagnosed Primary Sjögren Disease: A Prospective Observational Inception Cohort Study |
| **Study design** | Prospective observational inception cohort study |
| **Authors** | Kemal Erol; Ezgi Akyıldız Tezcan; Naciye Baş Bilen; Cemal Gürbüz |

| **Section** | **Item No.** | **Recommendation** | **Reported location / response in revised manuscript** |
| --- | --- | --- | --- |
| Title and abstract | 1(a) | Indicate the study’s design with a commonly used term in the title or the abstract. | Reported in the revised Title and Abstract/Methods: the manuscript is identified as a prospective observational inception cohort study. |
| Title and abstract | 1(b) | Provide in the abstract an informative and balanced summary of what was done and what was found. | Reported in the revised Abstract: objective, design, sample size, follow-up, adherence assessment, clinical and patient-reported measures, BMQ/NCD findings, exploratory logistic regression, and cautious non-causal conclusion are summarized. |
| Introduction | 2 | Explain the scientific background and rationale for the investigation being reported. | Reported in the Introduction: pSjD burden, medication adherence in chronic rheumatic diseases, limited pSjD-specific adherence data, previous Rheumatology International evidence on hydroxychloroquine blood levels, and the rationale for evaluating medication beliefs are described. |
| Introduction | 3 | State specific objectives, including any prespecified hypotheses. | Reported at the end of the Introduction: the study aimed to evaluate medication adherence during follow-up in newly diagnosed pSjD and examine associations with clinical characteristics, patient-reported outcomes, and beliefs about medicines. |
| Methods | 4 | Present key elements of study design early in the paper. | Reported at the beginning of Materials and Methods: prospective observational inception cohort design, consecutive enrollment, newly diagnosed pSjD, tertiary rheumatology outpatient clinic, and follow-up assessment are described. |
| Methods | 5 | Describe the setting, locations, and relevant dates, including periods of recruitment, exposure, follow-up, and data collection. | Reported in Materials and Methods/Study population and setting: tertiary hospital rheumatology outpatient clinic, Selçuk University Faculty of Medicine, Konya, Türkiye; baseline enrollment of newly diagnosed pSjD patients; follow-up data collection after a mean of 21.36 ± 4.79 months; ethics approval date and protocol details are provided in the Ethics section. |
| Methods | 6(a) | Give the eligibility criteria, and the sources and methods of selection of participants. Describe methods of follow-up. | Reported in Materials and Methods/Participants: adults aged 18–65 years with newly diagnosed pSjD fulfilling the 2016 ACR/EULAR classification criteria were enrolled consecutively. Follow-up assessment, scheduled visits, telephone contact, and subsequent in-person follow-up are described. |
| Methods | 6(b) | For matched studies, give matching criteria and number of exposed and unexposed. | Not applicable. This was not a matched cohort study. |
| Methods | 7 | Clearly define all outcomes, exposures, predictors, potential confounders, and effect modifiers. Give diagnostic criteria, if applicable. | Reported in Materials and Methods: diagnostic criteria for pSjD, adherence status based on CQR-5, ESSDAI, ESSPRI, HADS, fatigue severity, HAQ, SF-12, BMQ subscales, NCD, and sociodemographic/clinical variables are defined. Potential predictors assessed in exploratory analyses are specified. |
| Methods | 8 | For each variable of interest, give sources of data and details of methods of assessment (measurement). Describe comparability of assessment methods if there is more than one group. | Reported in Materials and Methods/Questionnaire-based assessments and clinical assessments: data sources, timing of assessment, instruments, scoring methods, validation status, and use of the same assessment procedures across adherence groups are described. |
| Methods | 9 | Describe any efforts to address potential sources of bias. | Reported in Materials and Methods and Limitations: consecutive enrollment, use of standardized instruments, comparison of follow-up completers with those lost to follow-up, cautious interpretation of self-reported adherence, and recognition of selection, recall, and social desirability bias are described. |
| Methods | 10 | Explain how the study size was arrived at. | Reported in Materials and Methods/Study size: the cohort comprised consecutive eligible newly diagnosed pSjD patients available within the study period; because this was an exploratory observational inception cohort, no formal a priori sample size calculation was performed. |
| Methods | 11 | Explain how quantitative variables were handled in the analyses. If applicable, describe which groupings were chosen and why. | Reported in Statistical Analysis: normality testing, mean ± SD or median (IQR), categorical variables as n (%), low/high adherence groups based on CQR-5, ESSDAI severity categories, ESSPRI categories, and change scores are described. |
| Methods | 12(a) | Describe all statistical methods, including those used to control for confounding. | Reported in Statistical Analysis: Shapiro-Wilk test, chi-square/Fisher exact tests, independent-samples t-test, Mann-Whitney U test, paired-samples t-test, Wilcoxon signed-rank test, and exploratory logistic regression with OR and 95% CI are described. Regression was limited because of small sample size. |
| Methods | 12(b) | Describe any methods used to examine subgroups and interactions. | Not applicable. No prespecified subgroup or interaction analyses were performed. |
| Methods | 12(c) | Explain how missing data were addressed. | Reported in Statistical Analysis/Results: analyses were performed using available follow-up data. Patients who completed follow-up and those lost to follow-up were compared to assess potential selection bias. No imputation was performed. |
| Methods | 12(d) | If applicable, explain how loss to follow-up was addressed. | Reported in Materials and Methods and Results: 40 of 54 patients completed follow-up; baseline characteristics of completers and patients lost to follow-up were compared, and the limitation related to loss to follow-up is discussed. |
| Methods | 12(e) | Describe any sensitivity analyses. | Not applicable. No formal sensitivity analyses were performed because of the exploratory design and small sample size. |
| Results | 13(a) | Report numbers of individuals at each stage of study—eg numbers potentially eligible, examined for eligibility, confirmed eligible, included in the study, completing follow-up, and analysed. | Reported in Results/Patient characteristics and adherence classification: 54 newly diagnosed pSjD patients were enrolled; 40 (74.1%) completed follow-up and were included in adherence analyses; 16 were classified as low adherence and 24 as high adherence. |
| Results | 13(b) | Give reasons for non-participation at each stage. | Reported where available in Results/Participants: 14 patients were lost to follow-up. Specific reasons for non-participation/loss to follow-up were not systematically recorded and are acknowledged as a limitation. |
| Results | 13(c) | Consider use of a flow diagram. | Participant flow is described in text. A flow diagram was considered but not included because the participant pathway was simple and the numbers are explicitly reported. |
| Results | 14(a) | Give characteristics of study participants (eg demographic, clinical, social) and information on exposures and potential confounders. | Reported in Results and Table 1: age, BMI, symptom duration, sex, employment, smoking, education, and baseline characteristics by adherence group are presented. |
| Results | 14(b) | Indicate number of participants with missing data for each variable of interest. | Reported in Results/Patient characteristics and Statistical Analysis: follow-up missingness is reported (14/54 lost to follow-up), and analyses were based on available data from the 40 reassessed patients. Variable-level missingness is reported where applicable in the revised manuscript. |
| Results | 14(c) | Summarise follow-up time (eg, average and total amount). | Reported in Results: mean follow-up duration was 21.36 ± 4.79 months. |
| Results | 15 | Report numbers of outcome events or summary measures over time. | Reported in Results and Tables 2–4: adherence categories at follow-up, ESSDAI/ESSPRI baseline and follow-up values, psychological symptoms, fatigue, HAQ, SF-12 values, BMQ scores, and NCD are reported. |
| Results | 16(a) | Give unadjusted estimates and, if applicable, confounder-adjusted estimates and their precision (eg, 95% confidence interval). Make clear which confounders were adjusted for and why they were included. | Reported in Results/Tables and Logistic regression: group estimates are reported for clinical and patient-reported variables. Exploratory logistic regression reports OR 1.95, 95% CI 1.01–3.76 for NCD. Multivariable adjustment was not performed because of the small sample size. |
| Results | 16(b) | Report category boundaries when continuous variables were categorized. | Reported in Materials and Methods/Results: CQR-5 low/high adherence classification, ESSDAI low/moderate/high categories, ESSPRI low/high symptom burden categories, and other categorical boundaries are described. |
| Results | 16(c) | If relevant, consider translating estimates of relative risk into absolute risk for a meaningful time period. | Not applicable. The main model was exploratory logistic regression for high adherence at follow-up, not a time-to-event or relative-risk model. |
| Results | 17 | Report other analyses done—eg analyses of subgroups and interactions, and sensitivity analyses. | Reported in Results: longitudinal change analyses and exploratory logistic regression are reported. No subgroup, interaction, or formal sensitivity analyses were performed. |
| Discussion | 18 | Summarise key results with reference to study objectives. | Reported at the beginning of Discussion: low adherence was frequent; adherence was not associated with disease activity or patient-reported burden but was associated with the necessity–concern differential. |
| Discussion | 19 | Discuss limitations of the study, taking into account sources of potential bias or imprecision. Discuss both direction and magnitude of any potential bias. | Reported in Limitations: small sample size, loss to follow-up, female predominance, self-report adherence bias, lack of detailed medication data, CQR-5 not specifically validated in pSjD, simultaneous assessment of adherence and BMQ, exploratory regression, and newly diagnosed versus symptom-duration heterogeneity are discussed. |
| Discussion | 20 | Give a cautious overall interpretation of results considering objectives, limitations, multiplicity of analyses, results from similar studies, and other relevant evidence. | Reported in Discussion and Conclusion: interpretation is cautious and non-causal; findings are discussed in light of previous rheumatic disease adherence studies and pSjD evidence, including Rheumatology International data. |
| Discussion | 21 | Discuss the generalisability (external validity) of the study results. | Reported in Limitations/Generalisability: single-center design, small sample size, female predominance, and newly diagnosed pSjD population may restrict generalisability; larger multicenter studies are recommended. |
| Other information | 22 | Give the source of funding and the role of the funders for the present study and, if applicable, for the original study on which the present article is based. | Reported in Declarations/Funding: this research received no specific grant from any funding agency in the public, commercial, or not-for-profit sectors; therefore, there was no funder role in study design, data collection, analysis, interpretation, or writing. |

**Information is provided separately for adherence groups where applicable, particularly in Tables 1–4 and the Results section.*
